# Decision-maker roles in healthcare quality improvement projects: a scoping review

**DOI:** 10.1136/bmjoq-2023-002522

**Published:** 2024-01-05

**Authors:** Justin Gagnon, Mylaine Breton, Isabelle Gaboury

**Affiliations:** 1 Department of Community Health Sciences, Université de Sherbrooke, Sherbrooke, Quebec, Canada; 2 Department of Family Medicine and Emergency Medicine, Université de Sherbrooke, Sherbrooke, Quebec, Canada

**Keywords:** Quality improvement, Decision making, Health policy, Leadership

## Abstract

**Objectives:**

Evidence suggests that healthcare quality improvement (QI) projects are more successful when decision-makers are involved in the process. However, guidance regarding the engagement of decision-makers in QI projects is lacking. We conducted a scoping review to identify QI projects involving decision-makers published in the literature and to describe the roles decision-makers played.

**Methods:**

Following the Joanna Briggs Institute framework for scoping reviews, we systematically searched for all types of studies in English or French between 2002 and 2023 in: EMBASE, MEDLINE via PubMed, PsycINFO, and the Cumulative Index to Nursing and Allied Health Literature. Criteria for inclusion consisted of literature describing health sector QI projects that involved local, regional or system-level decision-makers. Descriptive analysis was performed. Drawing on QI and participatory research literature, the research team developed an inductive data extraction grid to provide a portrait of QI project characteristics, decision-makers’ contributions, and advantages and challenges associated with their involvement.

**Results:**

After screening and review, we retained 29 references. 18 references described multi-site projects and 11 were conducted in single sites. Local decision-makers’ contributions were documented in 27 of the 29 references and regional decision-makers’ contributions were documented in 12. Local decision-makers were more often active participants in QI processes, contributing toward planning, implementation, change management and capacity building. Regional decision-makers more often served as initiators and supporters of QI projects, contributing toward strategic planning, recruitment, delegation, coordination of local teams, as well as assessment and capacity building. Advantages of decision-maker involvement described in the retained references include mutual learning, frontline staff buy-in, accountability, resource allocation, effective leadership and improved implementation feasibility. Considerations regarding their involvement included time constraints, variable supervisory expertise, issues concerning centralised leadership, relationship strengthening among stakeholders, and strategic alignment of frontline staff and managerial priorities

**Conclusions:**

This scoping review provides important insights into the various roles played by decision-makers, the benefits and challenges associated with their involvement, and identifies opportunities for strengthening their engagement. The results of this review highlight the need for practical collaboration and communication strategies that foster partnership between frontline staff and decision-makers at all levels.

**WHAT IS ALREADY KNOWN ON THIS TOPIC**

- Existing literature underscores the crucial role of decision-makers in the success of healthcare quality improvement (QI) projects; however, literature detailing their engagement and contributions is scant.

**WHAT THIS STUDY ADDS**

- This scoping review provides a comprehensive examination of QI projects involving decision-makers, highlighting their diverse roles and the specific advantages and challenges associated with their involvement.

**HOW THIS STUDY MIGHT AFFECT RESEARCH, PRACTICE OR POLICY**

- Insights from this review can guide the development of strategies for optimising decision-maker engagement in QI projects, potentially enhancing the success and sustainability of such initiatives.

## Introduction

Over the last decade, the use of quality improvement (QI) models in healthcare has become increasingly widespread. Health system decision-makers and frontline staff have embraced QI as a way of meeting the demand for increasing the quality, efficiency and cost-effectiveness of health service delivery.^1 2^ QI involves the systematic examination of processes and the development, implementation and evaluation of small-scale interventions, using rapid-cycle testing.^3^ It is, therefore, well-suited for rapidly adapting to changes in systemic and organisational conditions, and better aligning services to meet population needs.

Despite the potential for QI to enhance health service delivery, its impact appears to be mixed.^2^ While some evaluations have reported substantial improvement through the conduct of QI projects, in many cases the anticipated change is seldom achieved or sustained.^4–8^ A systems approach, taking into account the complexity and adaptability of healthcare systems,^9^ and involving frontline staff and decision-makers, is considered critical to successfully achieving and sustaining organisational transformation.^10–12^ Indeed, evaluations of QI projects have consistently highlighted the vital role of decision-makers in ensuring their success.^1 10 13–15^

Support and engagement from decision-makers at all levels are essential for staff buy-in and commitment to QI projects and promoting a culture of continuous improvement.^1 14 16 17^ In this paper, ‘decision-makers’ is used to signify actors who formally possess decisional^18^ (strategic, tactical or operational) authority and contribute to resource allocation within the organisational hierarchy. We distinguish between local decision-makers and regional decision-makers, whereby the former refers to organisational managers and administrators (eg, unit managers, frontline managers, hospital administrators, clinic directors), and the latter refers to regional administrators and policy-makers.^19^ At the organisational level, local decision-makers play a pivotal role in ensuring the delivery of high-quality care and patient safety. Their mandates include overseeing the implementation of effective strategies and practices that contribute to positive health outcomes.^15^ They possess the authority to expedite or impede the implementation of innovations within their organisation.^12 17^

In the context of QI projects, the support of decision-makers becomes particularly vital, especially when staff encounter time constraints and limited scheduling flexibility.^20^ Local decision-makers can address these challenges by actively championing the allocation of necessary resources and protected time for education and implementation activities.^21^ Furthermore, they can act as information brokers, facilitating communication and collaboration between different levels of management, and they can help bridge the gap between frontline staff and senior management, advocating for frontline staff’s needs and concerns.^12^ Moreover, local decision-makers can ensure the alignment of QI projects with organisational strategy, translate strategic priorities into actionable tasks, and delegate responsibilities.^12 20 22^ This strategic alignment, as well as their greater accountability to ensure the project is well-resourced and supported, can enhance the effectiveness and sustainability of QI projects.^12 17^ In addition to local decision-makers, regional and policy decision-makers also play a crucial role in supporting frontline staff’s participation in QI activities by establishing guidelines, providing funding opportunities and creating incentives that encourage frontline staff’s engagement in improvement efforts.^23 24^

Inclusion of decision-makers is not only considered critical to the success of QI projects, but evidence suggests that their continuous involvement throughout the QI project, beyond the initial stages, is associated with a higher probability of sustaining improvements.^22^ However, several authors have observed that decision-makers’ involvement seldom extends beyond an expression of support.^17 20^ By being actively engaged, decision-makers are better able to address challenges and make timely decisions throughout the process, provide ongoing guidance and support when issues arise, and ensure alignment of the project with organisational priorities.^6^ Securing their successful engagement requires a systematic strategy for communication and collaboration between decision-makers, frontline staff and other stakeholders involved in the QI project.^25^

Despite widespread recognition of the significance of decision-makers’ input and support and the need for their sustained engagement, there appears to be a dearth of literature focusing on their engagement and collaboration in QI projects.^16 26^ While QI teams would benefit from guidance on effectively involving decision-makers in these endeavours, authors report that QI reports tend to lack sufficient detail on how QI teams engaged with decision-makers^15 17^ and empirical research exploring the roles of decision-makers in QI implementation remains scant.^15 17 26^ To optimise decision-makers’ collaborative contributions and ensure the success and sustainability of QI projects, a deeper understanding of their engagement in QI activities is needed.

### Objectives

The objectives of this scoping review were to identify, within published literature, QI projects that involved decision-makers and describe the roles decision-makers played throughout. The review question was the following: what is the state of the literature regarding decision-makers’ involvement in healthcare QI projects?

## Methods

We conducted a scoping review following the Joanna Briggs Institute methodological framework^27^ and adhered to the Extension for Scoping Reviews of Preferred Reporting Items for Systematic Reviews and Meta-Analyses: Checklist and Explanation.^28^

### Search strategy

The search strategy (online supplemental appendix I) was collaboratively developed by the research team, with the assistance of a medical librarian. Literature search strategies were developed using medical subject headings and text words related to *quality improvement*^29^ and *decision-makers*. We searched Medline via Ovid, Embase via Ovid, PsycInfo via Ovid, and the Cumulative Index to Nursing and Allied Health Literature via EBSCO. We also searched ProQuest for relevant theses and dissertations, and the Institute for Healthcare Improvement for QI reports. However, an initial scan of these sources did not detect any eligible references, as decision-makers’ involvement was not sufficiently documented. Therefore, these sources were excluded from the search. Searches were restricted to English or French language texts published between 2002 and 2022, to focus on more recent literature. The search was conducted in April 2022 and updated in June 2023.

10.1136/bmjoq-2023-002522.supp1 Supplementary data

Our inclusion criteria specified that the literature should describe healthcare QI projects involving local, regional or system-level decision-makers. To be considered a QI project, the criteria for ‘continuous quality improvement’ (CQI) established by Rubenstein *et al*^30^ were used, which included: systematic data-driven activity, iterative development and testing, and adaptation to local conditions.^30^ We restricted the review to CQI as our study aim involved understanding decision-makers’ sustained engagement in improvement activities. There were no restrictions concerning the type of healthcare setting or the geographical location. References that did not describe, in detail, the processes or outcomes surrounding the conduct of a single QI project were excluded. References that did not mention decision-maker involvement during the abstract review stage were also excluded, unless projects were conducted in multiple sites, as we considered these to have a high probability of involving regional decision-makers.

### Study selection and data abstraction

Bibliographic data were imported into DistillerSR,^31^ whereupon duplicate entries were detected and removed. The titles and abstracts were then screened by two independent reviewers for eligibility. After samples of abstracts were reviewed, inclusion criteria were discussed and iteratively refined by the reviewers and research team. References about which reviewers disagreed were included for full-text screening. A complete dual review screening process was used at the title and abstract stage to minimise the risk of excluding an eligible reference.^32^

Following the abstract screening, the research team reviewed a sample of texts to identify references that met the established criteria for retention, thereby cultivating a common understanding and facilitating the identification of pertinent projects during the full-text review. The first author then screened the retained full texts. In cases where there was ambiguity regarding the exclusion of a reference, the senior author screened the reference and its inclusion was discussed.

Descriptive analysis^33^ of the data extracted from the retained full texts was performed. In the context of scoping reviews, descriptive analysis involves a comprehensive review and categorisation of collected literature. Given the exploratory nature of this study, descriptive analysis was an ideal analytic approach as it enabled us to map the landscape of existing literature, understand the range and depth of the topic and identify knowledge gaps that warrant future investigations.

The research team collaboratively developed an inductive data extraction grid, following an iterative process. This grid aimed to capture relevant QI project characteristics and the different roles decision-makers played. We examined published systematic reviews involving QI,^34–36^ and included fields that describe the QI projects (eg, objectives, intervention, QI framework, improvement outcome). To generate fields that met our objective and adequately reflected decision-makers’ roles and engagement (eg, roles, contributions, engagement strategies, benefits of collaboration and collaboration challenges), we drew on our experience with participatory research and QI. We categorised decision-makers’ contributions based on the extent of their involvement and the flow of information using a conceptual framework we developed following a deductive-inductive approach. Deductively, our framework combined elements adapted from participatory frameworks,^37 38^ including the IAP2’s Spectrum of Public Participation and the Steering Committee for Humanitarian Response’s Peer Review on Participation, which delineate varying degrees of involvement from information sharing to collaboration. We also drew on leadership and decision-making models,^39 40^ which describe the direction and support that leaders offer to their team members, from telling (authoritative) to delegating (collaborative). Inductively, our categorisation was influenced by our interpretation of the extracted data on decision-makers’ contributions to each of the projects (see online supplemental appendix III for examples) and enriched by QI literature detailing leadership roles in QI projects.^41^

10.1136/bmjoq-2023-002522.supp3 Supplementary data

Our conceptual framework describing decision-makers’ roles comprised four categories: initiator, supporter, consultant and collaborator. Initiators are decision-makers who spearhead QI projects, playing a role in strategic planning, preparation for implementation, identification of project champions, recruitment, delegation and the development of key project indicators. Supporters offer essential backing to QI projects, serving as advocates and sponsors, and providing implementation support. Consultants lend expertise to QI projects, assisting in the delineation of strategies, interventions and outcomes, to ensure alignment with organisational objectives. Collaborators, typically members of QI teams, actively engage in various project aspects including planning, implementation, team leadership, capacity building and the facilitation of change management.

Retained full texts were imported into NVivo (V.1.7)^42^ for coding and data extraction. Using NVivo, the first author tagged specific text selections from the retained references according to the initial coding grid. The research team then reviewed the outputs from the initial grid and generated a revised version that was used to complete the data extraction and analysis. Descriptive data were then inserted into tables to facilitate description and enable visual comparison and interpretation.

## Results

**Figure 1:**
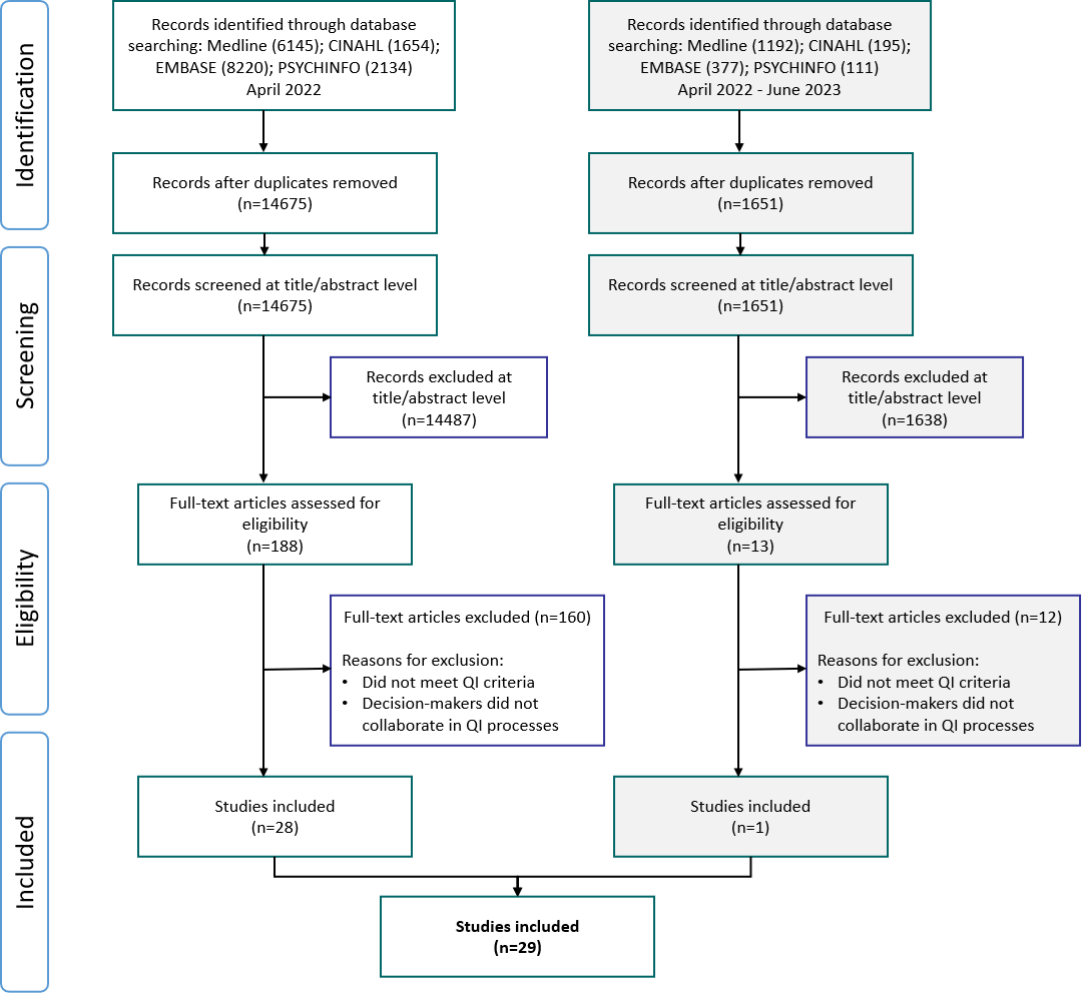
Extension for Scoping Reviews of Preferred Reporting Items for Systematic Reviews and Meta-Analyses diagram. QI, quality improvement.

As displayed by the study flow diagram (figure 1), our initial search (performed in April 2022) identified 14 675 references with duplicates removed using DistillerSR. After screening the titles and abstracts, 188 references remained. At this stage, we excluded 14 487 references that did not pertain to healthcare, did not involve QI projects, or did not mention the involvement of decision-makers in any capacity. We then proceeded to review the full texts of the remaining 188 references for inclusion. Of these, 28 met our criteria and were retained for data extraction. The 160 references were not retained due to either the criteria for CQI not being met or the lack of involvement of decision-makers. On re-running the search in June 2023, one additional reference was retained after the results were reviewed. Below, the data from these texts are presented according to QI context and processes, and engagement of decision-makers. The descriptive data are presented in full in tables 1 and 2 (expanded versions are provided in online supplemental file 2).

10.1136/bmjoq-2023-002522.supp2 Supplementary data

**Table 1:** Quality improvement (QI) project characteristics

Authors	Country	Setting (number)	Problem	Improvement framework	QI objective
Arbour *et al*^43^	USA	Primary care (5)	Maternal depression, intimate partner violence (IPV) and social needs	PDSA cycles	In five sites, 75% of infants and their families will receive all recommended well-child visits on time
Brush *et al*^44^	USA	Hospital (1)	Suboptimal cardiac surgery outcomes	None described	Improve cardiac surgery outcomes through practice standardisation
Chowdhury *et al*^58^	UK	Specialised (1)	Service users or their carers feel they are not given enough information	PDSA cycles	Improve communication between mental health team and service users, families and carers
Davies *et al*^45^	Ireland	Hospital (1)	Inefficiencies in nursing care and suboptimal work conditions	LSS	Optimise nursing time and improve personalised care and staff satisfaction
Doherty *et al*^46^	South Africa	Multiple institutions in one district	High rates of mother-to-child HIV transmission	None described	Scale up programme to prevent mother-to-child HIV transmission
Gage *et al*^47^	Zimbabwe	Multiple institutions (30) in five districts	Suboptimal maternal, newborn and child health services	PDSA cycles	Improve the quality of maternal, newborn and child health services
Gerrish *et al*^48^	UK	Multiple institutions	Risk of falls among elderly population and fall-related injury	Clinical micro-systems approach	Improve quality of care within the falls pathway
Haraden and Leitch^71^	Scotland	Hospital (9)	High rate of surgical mortality	Model for improvement (PDSA cycles)	Improve care quality (person-centred, safe and effective) for acute care patients
Hill *et al*^49^	USA	Hospital (1)	High rate of staff injury on inpatient child/adolescent psychiatric unit	I2S2 System of profound knowledge PDSA cycles	Reduce staff injury related to patient interactions in inpatient child/adolescent psychiatric unit
Iyengar *et al*^50^	India	Multiple institutions (44) in 10 districts	Gaps in the quality of childbirth services for institutional deliveries	None described	Improve quality of childbirth services public service facilities
Jaribu *et al*^51^	Tanzania	Multiple institutions (27) in two regions	Elevated maternal and neonatal morbidity and mortality	Breakthrough series collaborative PDSA cycles	Increase the rate of facility-based deliveries and improve the quality of perinatal care
Magge *et al*^70^	Ethiopia	Multiple institutions in four regions	Elevated rate of maternal and infant mortality	PDSA cycles	Improve maternal and child health and reduce maternal and infant mortality
Mahomed *et al*^52^	South Africa	Hospital (8)	High rate of healthcare associated infections	PDSA cycles	Implement a paper-based surveillance system for ICUs to measure healthcare-associated infections
Mate *et al*^53^	South Africa	Multiple institutions (161) in 18 health districts	High rate of mother-to-child HIV transmission	Model for improvement (PDSA cycles)	Improve the quality of antenatal care and prevent mother-to-child HIV transmission
Meehan *et al*^54^	USA	Specialised facilities (5)	High rate of hospital readmissions within 30 days postdischarge	None described	Decrease the rate of avoidable hospital readmissions
Needleman *et al*^55^	USA	Hospital (67)	Suboptimal safety, reliability and patient-centredness of inpatient care	PDSA cycles	Improve quality and efficiency of inpatient care and support more effective teamwork
Nyström *et al*^56^	Sweden	Specialised facilities in two municipalities	Suboptimal elder care and care for children with functional impairments	SIDSSA	Improve care knowledge and capabilities of service providers
Parikh *et al*^57^	USA	Hospital (1)	Inefficiencies in ambulatory surgery	Six Sigma (DMAIC)	Increase patient satisfaction, improve quality of care and increase efficiency of patient flow related to ambulatory surgery
Radwan *et al*^59^	UK	Primary care (1)	Inequity in the diabetes care and diabetes outcomes	PDSA cycles	Improve measurable diabetes-clinical outcomes in marginalised ethnic communities
Raman *et al*^60^	USA	Hospital (1)	Inefficiencies in adolescent scoliosis operations	None described	Improve perioperative efficiency in adolescent idiopathic scoliosis
Sermersheim *et al*^62^	USA	Hospital (1)	High incidence of hospital acquired injuries related to devices placed around the nares	‘RUSH Way’ (combines PDCA, Six Sigma, Lean)	Reduce the incidence of nares acquired pressure injuries (NAPIs) to 3%
Rocker *et al*^61^	Canada	Multiple institutions (78) across 10 provinces	Suboptimal COPD care and burden of disease for patients and health systems	PDSA cycles	Improve COPD care quality and patient-centredness
Rubenstein *et al*^69^	USA	Primary care (3) in three regions	Suboptimal quality of depression care	PDSA cycles	Implement research-based collaborative care for depression
Shi *et al*^68^	Taiwan	Hospital (1)	Elevated rate of surgical site infections	Six Sigma (DMAIC)	Reduce the surgical site rates by 20%
Taylor *et al*^63^	UK	Multiple institutions (10)	Suboptimal breast cancer care	None described	Improve multi-disciplinary team breast cancer care
Villarreal *et al*^64^	USA	Hospital (1)	Delays in procedure start times in radiology	None described	Improve efficiency and reduce delays in radiology start times
Waiswa *et al*^65^	Uganda	Hospital (6)	High rate of adverse birth outcomes	PDSA cycles	Improve the quality of maternal and neonatal care in hospitals
Welch *et al*^66^	USA	Hospital (1)	Burden of medically complex patients in neonatal intensive care units, and diminished collaboration and care continuity	PDSA cycles	Improve collaboration and continuity of care, decrease length of stay and improve parent satisfaction
Yapa *et al*^67^	South Africa	Primary care (7)	High rate of mother-to-child HIV transmission	PDSA cycles	Improve antenatal HIV care

COPD, chronic obstructive pulmonary disease; DMAIC, Define, Measure, Analyse, Improve, Control; ICU, intensive care unit; I2S2, Intermediate Improvement Science Series; LSS, Lean Six Sigma; PDSA, plan, do, study, act; SIDSSA, Sustainable Improvement and Development through Strategic and Systematic Approaches.

**Table 2:** The roles decision-makers played in the QI projects

Authors	QI project initiator	Local QI project leader(s)	Decision-maker level	Decision-makers’ roles and contributions
Arbour *et al*^43^	Non-government organisation	Local QI team	Regional	Insufficient detail
			Local	Clinic administrator: collaborator Member of local implementation team
Brush *et al*^44^	Local actor(s)	Local QI team	Local	Administrators: collaborator Member of Data Committee and Quality Committee, which initiated and led the project
Chowdhury *et al*^58^	Not specified	Local decision-maker	Local	Team manager, deputy manager and administrative manager: collaborators Participated in weekly meetings and PDSA cycles
Davies *et al*^45^	Not specified	Local QI team	Local	Hospital CEO and director of nursing (supporter and consultant) Project sponsor, consulted about the strategy
Doherty *et al*^46^	Regional decision-maker(s)	Regional QI teams	Regional	District programme managers: collaborator Participated in assessment, training workshops, feedback sessions, implementation and monitoring
			Local	Unit managers and clinic supervisors: collaborator Participated in assessment, training workshops, feedback sessions, implementation and monitoring
Gage *et al*^47^	Regional decision-maker(s)	Local QI team	Regional	Ministry of Health and Child Care: initiator Initiated the national programme, made QI a priority District managers: supporter Trained and supported to implement QI
			Local	Managers: collaborator Led the programme within each of the facilities
Gerrish *et al*^48^	Non-government organisation	Local QI team	Regional	Insufficient detail
			Local	Clinical managers: collaborator Member of meso-level and macro-level implementation groups Service managers: collaborator Involved in macro-level group for the achieving change phase
Haraden and Leitch^71^	Regional decision-maker(s)	Local decision-maker	Regional	Local health boards: supporter Supported clinician buy-in, participated in leadership walk-arounds Scottish overnment: initiator Contributed to measurement plan
			Local	Managers: collaborator Led local QI projects
Hill *et al*^49^	Not specified	Local QI team	Local	Nursing director: collaborator Member of QI team
Iyengar *et al*^50^	Regional decision-maker(s)	Not specified	Regional	State government: initiator Recruit of participants District health officers and state managers: supporter Provided report cards on project progress
			Local	Not specified
Jaribu *et al*^51^	Regional decision-maker(s)	QI facilitator	Regional	District managers: collaborator Participated in QI workshops, named ‘collaborators’
			Local	Insufficient detail
Magge *et al*^70^	Regional decision-maker(s)	Local QI team	Regional	Federal Ministry of Health: initiator Involved in improvement target and project selection, initiated and planned the initiative at the national level
			Local	Facility managers: collaborator Multidisciplinary team members
Mahomed *et al*^52^	Researchers	Researchers	Regional	Department of Health Provincial Infection Prevention and Control Unit and senior management: supporter Provided support to QI teams
			Local	Senior management and clinical manager: consultant Consulted for planning and intervention approval Nursing manager: consultant and supporter Consulted for planning, intervention approval, provided feedback, supported training
Mate *et al*^53^	Regional decision-maker(s)	Project leader	Regional	National Department of Health: initiator Initiated the national strategy District managers: supporter Designated participants, planned the project, participated in district review meetings
			Local	Facility managers: insufficient detail Involved in local execution of the QI project
Meehan *et al*^54^	Non-government organisation	QI facilitators	Regional	Not described
			Local	Administrators and nursing managers: supporter Asked for support, attended QI training, offered technical assistance
Needleman *et al*^55^	Non-government organisation	Local QI team Nursing unit	Regional	Insufficient detail
			Local	Unit managers: collaborator Attended or led the QI meetings, some were members of the QI leadership team Administrators and department heads: collaborator (some sites) Some participated in hospital level teams, some were members of the QI leadership team
Nyström *et al*^56^	Regional decision-maker(s)	Local decision-maker(s)	Regional	Division managers: collaborator Participated in meetings and workshops, oversaw change and implementation support
			Local	Unit managers: collaborator Participated in meetings and workshops, oversaw change and implementation support
Parikh *et al*^57^	Not specified	Local decision-maker(s)	Local	Administrators: collaborator Member of the leadership team
Radwan *et al*^59^	Regional decision-maker	Local QI team	Local	Managers: collaborator Codesigned the interventions, participated in QI training, implementation team member, facilitated reviews
Raman *et al*^60^	Local actor(s)	QI facilitator	Local	Senior executives and nurse managers: collaborator Provided training, participated in QI meetings, helped align goals, assessed needs, developed the intervention
Sermersheim *et al*^62^	Local actor(s)	Local QI team	Local	Nursing manager: collaborator Member of the QI team, codesign the project, participated in practice evaluation
Rocker *et al*^61^	Non-government organisation	Local QI team	Regional	Insufficient detail
			Local	Nurse managers: collaborator Member of local QI team
Rubenstein *et al*^69^	Researchers	Researchers	Regional	Regional director: collaborator Endorsed the project, participated in intervention design and in PDSA cycles
			Local	Clinical and administrative leaders: collaborator Participated in implementation and PDSA cycles
Shi *et al*^68^	Local actor(s)	Local QI team	Local	Managers at the general affairs office: collaborator Member of the QI team Dean and hospital administrators: supporter Approved improvement processes, supported implementation, provided the necessary resources
Taylor *et al*^63^	Regional decision-makers	Local decision-maker	Regional	Integrated care system (ICS) administrator: initiator and supporter Introduced the programme, received weekly updates
			Local	Managers and administrators: initiator and supporter Introduced the programme, identified champions, received weekly updates
Villarreal *et al*^64^	Local actor(s)	QI facilitator	Local	Manager and nurse manager: collaborator Member of the QI team, participated in assessment, planned and implemented interventions
Waiswa *et al*^65^	Non-government organisation	Local QI team	Regional	Health system managers, district health officers, minister of health obstetricians: collaborator Participated in health system managers meetings, codesigned the intervention, reviewed findings, assisted with implementation
			Local	Medical superintendents, hospital administrators, unit managers: collaborator Participated in health system managers meetings, assisted with intervention selection, reviewed findings, assisted with implementation
Welch *et al*^66^	Local actor(s) Clinician researchers	Local QI team	Local	Medical director of the NICU: consultant Consulted on the intervention and outcomes NICU nurse manager: collaborator Participated in QI meetings, collaborated on intervention development, reviewed results, proposed modifications
Yapa *et al*^67^	Non-government organisations	QI facilitators	Regional	Insufficient detail
			Local	Clinic operational managers: collaborator Provided guidance regarding the selection of improvement targets, some attended QI team meetings

NICU, neonatal intensive care unit; PDSA, plan, do, study, act; QI, quality improvement.

### QI context and processes

Table 1 displays the characteristics of the QI projects as described in the 29 references. 26 references^43–68^ provided a retrospective description of QI processes and a longitudinal evaluation of their impact on selected outcomes, and three^69–71^ reported findings at an intermediary stage. Among the selected references, four^47 48 52 54^ also reported on factors that either facilitated or hindered the implementation of the QI programme, and five^55 56 61 63 70^ focused primarily on outcomes related to implementation measures.

The QI projects were distributed across different regions, with twelve projects taking place in North America, nine in Africa, seven in Europe and one in Asia. A total of 18 projects^43 46–48 50–55 61 63 65 67 69–71^ were implemented across multiple sites, while 11 projects^44 45 49 57–60 62 64 66 68^ were carried out at a single site. The QI projects were implemented in various healthcare settings, including hospitals (n=13),^44 45 49 52 55 57 60 62 64 66 68 71^ primary care clinics (n=4),^43 59 67 69^ specialised care centres (n=4),^54 56 58 61^ or a combination of different facilities within or across health districts (n=8).^46–48 50 51 53 63 70^ These projects addressed a diverse range of problems, reflecting the varied focus areas of QI efforts. Out of the 29 references, 22 discussed the utilisation of a QI framework to facilitate the rapid testing of small-scale interventions. Among these, 15 teams^43 47 51–53 55 58 59 61 65–67 69–71^ employed PDSA (‘plan, do, study, act’) cycles, three^45 57 68^ followed the Six Sigma framework, one^56^ used SIDSSA (‘Sustainable Improvement and Development through Strategic and Systematic Approaches’) cycles, one^48^ employed a clinical microsystems approach and two^49 62^ adapted elements from various frameworks. The interventions developed to address gaps in healthcare, whether at a local or large-scale level, exhibited a range of approaches.

Table 1 also provides an overview of the decision-makers involved in the QI projects, categorised as either local (eg, managers and directors) or regional (eg, district managers, regional administrators and national policy decision-makers) actors. Out of the 29 projects, 17 described the involvement of local decision-makers exclusively, 10 projects involved both regional decision-makers and local decision-makers, and 2 projects described the involvement of only regional decision-makers, not local decision-makers. Looking at the 18 multi-site projects, 10 described the involvement of both regional and local decision-makers, while 4 focused on local decision-makers and 2 focused only on regional decision-makers.

### Involvement of decision-makers

Table 2 describes the characteristics of the papers in terms of the roles of various actors and especially the involvement of decision-makers. Regarding the initiation of the QI project, we observed a diverse range of actors involved. Notably, out of the 18 multi-site projects, regional decision-makers initiated 10,^46 47 50 51 53 56 59 63 70 71^ while non-government partners (either academic or health professional entities) or QI organisations initiated 7.^43 48 54 55 61 65 67^ Among the 11 single-site projects, local actors, such as QI teams or health professionals, initiated 6 projects.^44 60 62 64 66 68^ In four instances, however, the references provided insufficient detail to determine who initiated the projects.^45 49 57 58^ We also observed a diverse range of actors involved in local project leadership. Local QI teams or teams of health professionals led 14 of the 29 projects,^43–45 47 48 55 59 61 62 65 66 68 70^ local decision-makers led 5,^56–58 63 71^ QI facilitators led 5,^51 54 60 64 67^ researchers led 2,^52 69^ a project leader led 1,^53^ regional QI teams led 1^46^ and 1 provided insufficient detail to determine.^50^

#### Decision-maker roles

In table 2, we also provide a comparison of the roles and contributions of local and regional decision-makers. This enabled our illustration of the degree of collaboration and the details provided regarding the nature of their collaboration. Local decision-makers’ contributions were documented in 27 of the 29 projects and regional decision-makers’ contributions were documented in 12. Based on these documented contributions and following the conceptual framework (described above), we categorised the roles played by regional and local decision-makers as: initiator, supporter, consultant and collaborator.

In 7 out of 12 projects,^47 49 50 53 63 70 71^ regional decision-makers were the initiators of the QI projects. Their activities included strategic planning and preparation for implementation^47 50 53 70^; recruitment, delegation and coordination of teams^50 63^; assessment and definition of indicators.^71^ Regional decision-makers played supportive roles in six projects.^45 47 50 52 63 71^ Their supportive activities consisted of advocacy and sponsorship^71^; implementation support^47 52^; and participation in review meetings and progress feedback.^50 53 63^ Regional decision-makers were not described as having played a consultative role in any of the projects. Finally, in 5 of 12 projects,^46 51 56 65 69^ regional decision-makers played a collaborative role, participating actively in QI activities^46 51 56 65 69^; and helping with implementation and change management.^46 56^

In most projects that described the involvement of local decision-makers, 22 of 27,^43 44 46–49 55–62 64–71^ they acted as collaborators and regular members of the QI team. They contributed to assessment and planning^62 64 65^; actively participated in QI activities^46 49 58 62 64^; led and coordinated teams^47 55 57 58 60 67 71^; helped with implementation and change management^46 56 64 65 69^; and supported capacity building.^59 60 62^ In three projects,^45 52 66^ local decision-makers were described as having been consulted for defining the strategy, intervention and outcomes. Their involvement included strategic planning and preparation for implementation^45 52 66^; assessment^52^; and approval of QI project components.^52^ Local decision-makers acted as supporters in five projects,^45 52 54 63 68^ engaging in advocacy and sponsorship^45^; offering technical assistance and support^54 68^; and providing training and skill development.^52 54^ Finally, in one project^63^ we considered local decision-makers to have played the role of initiator, in which they were described as having introduced the QI programme and identified project champions.

#### Engagement strategies, advantages of collaboration and challenges

None of the references explicitly described a strategy for including decision-makers as consistent collaborators throughout the QI projects, and only one of the references^48^ employed a QI framework intended to foster stakeholder engagement (ie, clinical microsystems approach). In many of the projects, local decision-makers were members of the QI team. The range of expected or observed benefits of involving decision-makers in QI projects, categorized as follows, were discussed in 15 of the 29 texts (supportive quotes provided in online supplemental appendix III).

- Promote cooperation and shared learning^44^: collaboration with decision-makers encouraged a spirit of cooperation and helped foster an environment conducive to shared learning, leadership and problem-solving.
- Enhance frontline staff buy-in^44 69 71^: involvement of decision-makers was considered pivotal in gaining the buy-in of frontline staff.
- Support decision-makers’ sense of ownership and accountability^46 59^: decision-makers involvement helped motivate their contribution toward the project’s success.
- Secure resources and support^45 70^: decision-makers play a role in advocating among senior management to secure financial investment. Decision-makers’ participation can also facilitate the enactment of policies that are favourable to QI.
- Enable more effective leadership^47 53 56 57 70^: decision-makers’ involvement can deepen their understanding of frontline staff perceptions and the operational realities of their facilities.
- Ensure feasibility and successful implementation^65 67^: decision-makers’ in-depth understanding of the organisation’s capabilities and constraints helps to ensure that projects are feasible and adapted to the organisation’s needs. Their endorsement (or authorisation) is considered to be critical.

In 14 of 29 references, (supportive quotes provided in online supplemental appendix III), the authors discussed challenges or identified facilitators or barriers to QI implementation that concerned decision-makers’ involvement or their roles. These are listed below.

- Time constraints and support from senior management^46 52 54 67^: frontline managers had time commitments preventing their participation in QI. Senior management support was considered critical in enabling frontline managers’ participation.
- Variable expertise and quality of supervision^47 50 56 61^: lack of managerial experience and training hindered optimal facilitation and implementation. Investment in the development of managerial skills and ensuring the provision of high-quality supervision and monitoring was considered essential.
- Centralised leadership^47 51 52 63^: highly centralised approaches potentially hinder the sense of ownership among frontline managers and staff and impact QI project acceptability. Balanced leadership that encourages active participation at all levels is recommended.
- Communication and stakeholder relationships^57 61 63^: poor stakeholder relationships can result in participation issues and contribute to the adoption of more centralised approaches. Regular and effective communication between stakeholders can enhance the success and sustainability of QI projects.
- Alignment of objectives and strategies^66^: discrepancies between managerial strategies and the QI project can hinder adequate adaptation of the project to the operational realities and the provision of required resources. Ensuring synergy between managerial and frontline staff’s priorities and implementation plans is considered essential.

The results of this scoping review provide insight into the range of roles decision-makers have played in QI projects and highlight the importance of their involvement in securing the project’s success. While the advantages such as mutual learning, frontline staff buy-in, accountability, resource allocation, effective leadership and feasible implementation are evident, issues related to time constraints, supervisory expertise, centralised leadership, stakeholder relationships and strategic alignment pose significant challenges. These findings set the stage for an in-depth discussion on their implications for the design and implementation of QI projects.

## Discussion

This scoping review was conducted with the objectives of identifying QI projects within published literature that involved decision-makers and describing the roles they played. We included decision-makers of all levels in our review and contrasted the roles played by local and regional decision-makers. To the best of our knowledge, this is the first scoping review to identify references that describe QI projects which include decision-makers. It was motivated by evidence suggesting that the success of QI projects often hinges on decision-makers’ continuous collaboration, and the apparent lack of literature discussing how they were successfully involved. While our review did not identify any formal engagement strategies in the texts we retained, it does provide important insights into decision-makers’ roles and advantages and challenges associated with their involvement and gives rise to recommendations for collaboration with decision-makers.

We observed variation between the roles of local and regional decision-makers. Regional decision-makers commonly initiated and supported QI projects, playing a role in strategic planning, capacity building, implementation support and feedback mechanisms. Occasionally, they acted as consultants or collaborators, in which they actively participated in QI processes and supported change management. Conversely, local decision-makers were predominantly collaborators, contributing significantly to planning, coordination, implementation, change management and capacity building. They also periodically functioned as consultants and supporters, engaging in strategic planning, assessments, technical support and skill development. While the variability in roles demonstrates adaptability in QI projects, suboptimal engagement frequently hinders successful and sustained implementation, as many authors have noted.^12 17 20^ As highlighted in our review, issues such as insufficient resources, limited time, and conflicting goals and strategies are obstacles to successful QI implementation. Furthermore, the quality of relationships between frontline staff and decision-makers is strained by poor communication, deficient QI support and non-collaborative leadership styles, which can exacerbate QI team disengagement.^47 51 57 63^ Addressing these challenges and leveraging the reported benefits of decision-maker engagement could significantly improve the effectiveness and sustainability of QI initiatives.

The results of this review point to the necessity of developing practical engagement and communication strategies that foster a spirit of collaboration between frontline staff, decision-makers at all levels and other stakeholders. Our review highlights the importance of adopting less centralised leadership styles that embrace input from stakeholders and foster a sense of ownership among all involved. Given recommendations in the literature that decision-makers play a collaborative role in QI projects and evidence suggesting their involvement is critical to success,^12 14 15 17 20^ we expected engagement strategies to be well documented. However, none of the references described engagement strategies, and few provided rationale for their involvement or a discussion of the impact of their involvement on implementation. The scarcity of documented engagement strategies points to a significant opportunity for future research. Future research should evaluate techniques for fostering ongoing engagement and effective communication and collaboration with stakeholders throughout the project lifecycle, and strategies for reconciling stakeholders’ different priorities and expectations. A deeper understanding of engagement mechanisms and collaboration strategies is essential for ensuring the success and sustainability of QI projects.

The review also revealed differences in the documentation of decision-maker involvement between single-site and multi-site QI projects. These differences primarily centred around the focus and level of detail provided. Single-site projects generally provided more detail about local processes and stakeholders, whereas multi-site projects tended to offer more detail about the larger QI initiative and provided less detail about how local teams functioned and collaborated. In multi-site projects, details regarding rapid testing of interventions were less often provided. The lack of uniformity in reporting between multi-site and single-site projects restricted our in-depth comparison of decision-makers roles across different types of QI projects. Our observations corroborate the assertion that more detailed documentation of local processes is needed regarding multi-site projects,^72^ so that we might gain insight into local decision-makers’ contributions considering these typically centralised implementation strategies.

### Limitations

This study encountered several limitations. First, in terms of data sources, we primarily focused our search within research journals. We searched several additional sources for QI reports, but we did not identify any text that met our criteria for the documentation of decision-makers’ involvement. We acknowledge that QI differs from traditional research and that the texts we retained may not fully reflect the spectrum of QI efforts in practice. The texts we included may be more skewed toward presenting QI in the context of research, such as evaluation of the outcomes of QI implementation, or identification of implementation barriers and facilitators. To capture a more comprehensive picture of QI efforts, broader search strategies that include additional grey literature sources may be warranted for future research. Second, our search strategy identified texts that used terms more commonly associated with QI (eg, ‘quality improvement’, PDSA, define-measure-analyse-improve-control, Lean, Six Sigma, rapid cycle, etc). Studies that did not employ this specific terminology might have been missed; however, we do not consider the number of missed studies to be significant. Third, to identify decision-makers, we employed several terms in our search strategy (eg, decision-maker, policy-maker, manager, administrator). We did not include terms such as ‘directors’, ‘ministers of health’, ‘CEOs’ or ‘health authorities’. As our search identified 14 675 references after duplicates were removed, broadening our search would not have been feasible. Finally, in our search for texts, we used the documentation of participatory research as a frame of reference, which typically emphasises stakeholders’ contributions. Consequently, we expected that abstracts would explicitly mention the involvement of decision-makers. However, given the importance of decision-makers’ involvement, we do not believe that those we excluded in this way would have involved the active participation of decision-makers.

## Conclusion

This scoping review provides important insights into decision-makers’ contributions to QI projects. Recognising their vital role in the success of these initiatives, this paper uncovers the various roles played by decision-makers, the benefits and challenges associated with their involvement, and identifies opportunities for strengthening their involvement. Addressing barriers such as limited resources, time constraints, and conflicting priorities, healthcare organisations can better capitalise on decision-makers’ participation in QI projects. We emphasise the need for practical engagement and communication strategies that foster collaborative stakeholder partnerships. Future studies should focus on developing and evaluating explicit and actionable strategies for engaging decision-makers in QI processes to further enhance QI outcomes.

## Data Availability

No data are available.
