# Supplementary material for: Decision-maker roles in healthcare quality improvement projects: a scoping review
Source: BMJ Open Qual. 2024 Jan 5;13(1):e002522. doi: 10.1136/bmjoq-2023-002522 (PMC10773379; doi:10.1136/bmjoq-2023-002522)
Supplement: Supplementary data [file bmjoq-2023-002522supp001.pdf]

Appendix I. Quality Improvement and Decision-Maker Search Strategy

MEDLINE (OVID)

#	Query	Results from April 4, 2022
1	"Quality improvement"/	30,574
2	(Quality adj3 improv*).tw.	183,201
3	(pdsa or plan-do-study-act or "plan do study act" or pdca or plan-do-check-act or "plan do check act" or define-measure-analyze-improve control or dmaic or dmadv or define-measure-analyze-design-verify).tw.	2,421
4	((lean adj manufacturing) or (lean adj production) or (lean adj healthcare) or (lean adj health adj care) or (lean adj health adj service) or (lean adj healthcare adj service) or (lean adj health adj care adj service) or (inventive adj problem adj solving) or (inventive adj problem-solving) or (inventive adj problem solving) or (business adj process adj reengineering) or (business adj process adj re-engineering) or (system* adj redesign)).tw.	604
5	((iterative adj cycle) or (rapid adj cycle) or (small adj test adj2 change)).tw.	655
6	(deming or taguchi or kansei or kaizen or (toyota adj production adj system)).tw.	2,177
7	(six-sigma or (six adj sigma) or "total quality management" or (quality adj function adj deployment) or (quality adj circle) or (quality adj cycle)).tw.	1,953
8	(policy-maker* or policymaker* or decisionmaker* or decision-maker*).tw.	61,920
9	(administrator* or manager*).tw.	81,017
10	1 or 2	198,463
11	3 or 4 or 5 or 6 or 7	7,514
12	10 or 11	203,451
13	8 or 9	137,897
14	12 and 13	7,218
15	limit 14 to yr="2002 -Current"	6,319
16	limit 15 to (english or french)	6,145

CINAHL (EBSCO)

#	Query	Results from April 4, 2022
S17	S12 AND S13 (English or French) AND 2002-2022, Exclude MEDLINE	1,654
S16	S12 AND S13 (English or French) AND 2002-2022	2,813
S15	S12 AND S13 (English or French)	3,068
S14	S12 AND S13	3,121
S13	S8 OR S9	82,864
S12	S10 OR S11	72,878
S11	S3 OR S4 OR S5 OR S6 OR S7	3,905
S10	S1 OR S2	70,964
S9	(TI administrator* OR AB administrator*) OR (TI manager* OR AB manager*)	56,724
S8	(TI policy-maker* OR AB policy-maker*) OR (TI policymaker* OR AB policymaker*) OR (TI decisionmaker* OR AB decisionmaker*) OR (TI decision-maker* OR AB decision-maker*)	28,735
S7	((TI six-sigma OR AB six-sigma)) OR ((TI six OR AB six) W1 (TI sigma OR AB sigma)) OR ((TI "total quality management" OR AB "total quality management")) OR ((TI quality OR AB quality) W1 (TI function OR AB function) W1 (TI deployment OR AB deployment)) OR ((TI quality OR AB quality) W1 (TI circle OR AB circle)) OR ((TI quality OR AB quality) W1 (TI cycle OR AB cycle))	1,445
S6	(TI deming OR AB deming) OR (TI taguchi OR AB taguchi) OR (TI kansei OR AB kansei) OR (TI kaizen OR AB kaizen) OR ((TI toyota OR AB toyota) W1 (TI production OR AB production) W1 (TI system OR AB system))	406
S5	((TI iterative OR AB iterative) W1 (TI cycle OR AB cycle)) OR ((TI rapid OR AB rapid) W1 (TI cycle OR AB cycle)) OR ((TI small OR AB small) W1 (TI test OR AB test) N2 (TI change OR AB change))	451
S4	((TI lean OR AB lean) W1 (TI manufacturing OR AB manufacturing)) OR ((TI lean OR AB lean) W1 (TI production OR AB production)) OR ((TI lean OR AB lean) W1 (TI healthcare OR AB healthcare)) OR ((TI lean OR AB lean) W1 (TI health OR AB health) W1 (TI care OR AB care)) OR ((TI lean OR AB lean) W1 (TI health OR AB health) W1 (TI service OR AB service)) OR ((TI lean OR AB lean) W1 (TI healthcare OR AB healthcare) W1 (TI service OR AB service)) OR ((TI lean OR AB lean) W1 (TI health OR AB health) W1 (TI care OR AB care) W1 (TI service OR AB service)) OR ((TI inventive OR AB inventive) W1 (TI problem OR AB problem) W1 (TI solving OR AB solving)) OR ((TI inventive OR AB inventive) W1 (TI problem-solving OR AB problem-solving)) OR ((TI inventive OR AB inventive) W1 (TI problem solving OR AB problem solving)) OR ((TI business OR AB business) W1 (TI process OR AB process) W1 (TI reengineering OR AB reengineering)) OR ((TI business OR AB business) W1 (TI process OR AB process) W1 (TI re-engineering OR AB re-engineering)) OR ((TI system* OR AB system*) W1 (TI redesign OR AB redesign))	444
S3	(TI pdsa OR AB pdsa) OR (TI plan-do-study-act OR AB plan-do-study-act) OR (TI "plan do study act" OR AB "plan do study act") OR (TI pdca OR AB pdca) OR (TI plan-do-check-act OR AB plan-do-check-act) OR (TI "plan do check act" OR AB "plan do check act") OR (TI define-measure-analyze-improve control OR AB define-measure-analyze-improve control) OR (TI dmaic OR AB dmaic) OR (TI dmadv OR AB dmadv) OR (TI define-measure-analyze-design-verify OR AB define-measure-analyze-design-verify)	1,376
S2	Quality N3 (TI improv* OR AB improv*)	17,187
S1	(MH "Quality improvement")	62,473

EMBASE (OVID)

#	Query	Results from April 4, 2022
1	"Quality improvement"/	48,631
2	(Quality adj3 improv*).tw.	261,291
3	(pdsa or plan-do-study-act or "plan do study act" or pdca or plan-do-check-act or "plan do check act" or define-measure-analyze-improve control or dmaic or dmadv or define-measure-analyze-design-verify).tw.	5,846
4	((lean adj manufacturing) or (lean adj production) or (lean adj healthcare) or (lean adj health adj care) or (lean adj health adj service) or (lean adj healthcare adj service) or (lean adj health adj care adj service) or (inventive adj problem adj solving) or (inventive adj problem-solving) or (inventive adj problem solving) or (business adj process adj reengineering) or (business adj process adj re-engineering) or (system* adj redesign)).tw.	788
5	((iterative adj cycle) or (rapid adj cycle) or (small adj test adj2 change)).tw.	936
6	(deming or taguchi or kansei or kaizen or (toyota adj production adj system)).tw.	3,149
7	(six-sigma or (six adj sigma) or "total quality management" or (quality adj function adj deployment) or (quality adj circle) or (quality adj cycle)).tw.	2,437
8	(policy-maker* or policymaker* or decisionmaker* or decision-maker*).tw.	68,414
9	(administrator* or manager*).tw.	82,217
10	1 or 2	280,240
11	3 or 4 or 5 or 6 or 7	12,583
12	10 or 11	287,770
13	8 or 9	145,407
14	12 and 13	9,088
15	limit 14 to yr="2002 -Current"	8,431
16	limit 15 to (english or french)	8,220

PSYCHINFO (OVID)

#	Query	Results from June 8 2023
1	"Quality improvement"/	0
2	(Quality adj3 improv*).ti,ab.	31,136
3	(pdsa or plan-do-study-act or "plan do study act" or pdca or plan-do-check-act or "plan do check act" or define-measure-analyze-improve control or dmaic or dmadv or define-measure-analyze-design-verify).ti,ab.	293
4	((lean adj manufacturing) or (lean adj production) or (lean adj healthcare) or (lean adj health adj care) or (lean adj health adj service) or (lean adj healthcare adj service) or (lean adj health adj care adj service) or (inventive adj problem adj solving) or (inventive adj problem-solving) or (inventive adj problem solving) or (business adj process adj reengineering) or (business adj process adj re-engineering) or (system* adj redesign)).ti,ab.	441
5	((iterative adj cycle) or (rapid adj cycle) or (small adj test adj2 change)).ti,ab.	102
6	(deming or taguchi or kansei or kaizen or (toyota adj production adj system)).ti,ab.	318
7	(six-sigma or (six adj sigma) or "total quality management" or (quality adj function adj deployment) or (quality adj circle) or (quality adj cycle)).ti,ab.	935
8	(policy-maker* or policymaker* or decisionmaker* or decision-maker*).ti,ab.	38,895
9	(administrator* or manager*).ti,ab.	96,895
10	1 or 2	31,136
11	3 or 4 or 5 or 6 or 7	1,973
12	10 or 11	32,729
13	8 or 9	131,306
14	12 and 13	2,511
15	limit 14 to yr="2002 -Current"	2,194
16	limit 15 to (english or french)	2,134
