# Supplementary material for: Decision-maker roles in healthcare quality improvement projects: a scoping review
Source: BMJ Open Qual. 2024 Jan 5;13(1):e002522. doi: 10.1136/bmjoq-2023-002522 (PMC10773379; doi:10.1136/bmjoq-2023-002522)
Supplement: Supplementary data [file bmjoq-2023-002522supp003.pdf]

Appendix III. Illustrative Examples and Quotes

Table 3. Illustrative Examples of Local and Regional Decision-Makers' Contributions by Type of Role

	Local Decision-Makers	Regional Decision-Makers
Initiator	"MDT-FIT was designed to be coordinated by cancer managers/administrators within individual hospitals. For this implementation study this role was undertaken by an ICS administrator (non-clinical), managed by the ICS Director."<br><br>"The ICS administrator was advised to visit each breast multidisciplinary team to introduce them to MDT-FIT and was also advised to identify a local […] 'champion' that could help support engagement and coordination in each hospital." [1]	"This intervention titled "Parijaat" was designed by Action Research & Training for Health, in partnership with the state government and United Nations Population Fund."<br><br>""Parijaat", emerged as a collaborative initiative for improving the quality of facility based delivery services as a result of consultations between representatives of the state government's Medical, Health & Family Welfare Department, UNFPA, UNICEF and Action Research & Training for Health (ARTH), a notfor-profit organization."<br><br>"The state government issued a letter to all participating facilities, positioning the intervention as being one for quality improvement." [2]
Supporter	"A QIO staff […] mailed a recruitment letter to each SNF Administrator or Director of Nursing. […] The letter emphasized the national focus on reduction of preventable hospital readmissions and Medicare's strategies […]. Next, 2 other QIO staff members […] placed follow-up phone calls […] to request a face-to-face meeting with the Administrator, the Director of Nursing, and the Medical Director to discuss the project, answer questions, and obtain leadership commitment to participate."<br><br>"Then, QIO personnel made 1-3 phone calls to the Directors of Nursing Services and the Administrators of each SNF to schedule on-site recruitment visits. Leaders at 7 facilities agreed to meet with QIO staff to discuss the project." [3]	"The implementation of the surveillance system for HAIs in ICUs received strong support from the KwaZulu-Natal Department of Health Provincial Infection Prevention and Control Unit and senior management at each of the hospitals." [4]
Consultant	"The intervention and primary outcomes for this project were developed initially in consultation with the medical director of the NICU." [5] Welch et al.	None described
Collaborator	"The stakeholders involved were the team manager and deputy manager, the team consultant, the team specialist registrar, team administrative manager, two carers and one service user. […]The team organised weekly meetings to brainstorm ideas, plan tests of change to review progress and to agree on the next course of action."<br><br>"The team attended the service user forum […] The team developed a theory of change as a driver diagram […] Plan, Do, Study, Act (PDSA) cycles involved the testing of posters in clinical spaces, follow-up phone calls to service users and the amendment of clinic letters.[…] The project team developed a forcefield analysis to identify the factors supporting and restraining people from adopting this change idea." [6]	"Participatory assessment phase - District programme managers gain skills in programme assessment:<br>• Training workshop on assessment framework and tools […]<br>• Formation of teams of 3-4 people<br>• Complete assessment of PHC facilities […]<br>• Collection of routine data […]"<br><br>"Feedback and planning phase - District managers identify areas of weakness and learn to set realistic targets and action plans:<br>• Review of assessment results at a workshop<br>• Identification of areas of weakness<br>• Target setting and action plans"<br><br>"Implementation and monitoring phase - Team agrees on an action plan to address programme weakness […]:<br>• Planned interventions implemented […]<br>• Development of further action plans" [7]

Table 4. Selected Excerpts Highlighting the Advantages of Decision-Maker Involvement in QI Projects

Advantages Described	Quote
Promote cooperation and shared learning	"Over the years, hospital administration and key physician leaders have managed to promote a sustained spirit of cooperation." [8]<br><br>"These committees [two committees consisting of high-level individuals to launch, coordinate and institutionalize CQI] offer an opportunity for shared learning, leadership, and problem solving." [8]
Enhance frontline staff buy-in	"Over the years, hospital administration and key physician leaders have managed to promote a sustained spirit of cooperation. The chair of each committee is physician, and the agenda is focused on clinical performance and not individual financial performance. The agenda of meetings primarily focuses on hospital wide performance and induces all parties to buy into the concept of improving overall systems of care." [8]<br><br>"To be successful, TIDES required support from both primary care and mental health leadership at multiple levels (region, medical center, and local practice). Buy-in from the managerial levels that control resource allocation was also critical" [9]<br><br>"Scotland's local health boards were recruited to convince hospital staff and patients that safety was a priority." [10]
Support decision-makers' sense of ownership and accountability	"The advantages of a participatory approach are that the process of conducting the assessments enables mid level managers to see first hand how well their facilities are functioning and to take ownership of the findings since it is data that they themselves have collected." [7]<br><br>"Clinical pharmacists, administrative assistants and practice managers played a role in the implementation and delivery. Overall, this QI programme enabled a sense of ownership by the whole practice team." [11]
Secure resources and support	"The involvement of senior management and their commitment to investing the initial costs of a LSS project were crucial to the progress of the project." [12]<br><br>"A critical factor in the successful implementation of the prototype phase has been the FMoH's leadership of the design and implementation at all levels. The impetus for program design came from the FMoH in alignment with the Quality and Equity transformation agenda laid out in Ethiopia's HSTP." [13]<br><br>"The deep engagement of the FMoH and incorporation of the QI strategy into central policy and planning were major guarantors of survival of the program during periods of instability and change." [13]
Enable more effective leadership	"Enabling factors included strengthened leadership, teamwork and joint decision-making at facilities, as well as and supportive supervision." [14]<br><br>"Two notable achievements—rapid buy-in and project leadership by district managers, and collaboration of multiple supporting NGO partners—were responsible for early success of the intervention and provide a potential model for implementation of other large-scale programs in similar resource-constrained settings." [15]<br><br>"In retrospect, including managers who were new in their role, and participants on the way to retirement, was not optimal. Nevertheless, strategic management involvement, coaching, and working with managerial colleagues enhanced the observed change and learning processes." [16]<br><br>"Proper leadership involving each stakeholder, obtaining active feedback, and clearly explaining the goals of the project are key to any successful endeavor." [17]<br><br>"Finally, we found learning sessions to be an effective way to link health system actors directly with community members to advance patient-centered care. During the last prototype learning sessions, community members vetted the change package and helped set priorities for future improvement activities." [13]
Ensure feasibility and successful implementation	"First, the designed interventions were feasible within the context because managers generally knew what would or would not work in their context. Second, the managers endorsed the interventions and thus were more willing to cooperate with the implementation and maintenance of those things that were deemed useful." [18]<br><br>"The operational manager was essential for decision-making to start a new activity." [19]

Table 5. Selected Excerpts Highlighting the Challenges of Involving Decision-Makers in QI Projects

Challenges Described	Quote
Time constraints and support from senior management	"The disadvantages are that it relies on support and buy in from senior district management to allow mid level managers the time to participate in the workshops and to actually undertake the assessments. Without this support, the participatory approach would not succeed." [7]<br><br>"Despite the intensive planning, stakeholder engagement, consultation and training of nursing staff, and support from management, the surveillance of HAIs in ICUs was not successful. The main reason for the failure of the surveillance could be attributed to human resource limitations." - [4]<br><br>"Leaders from the 8 SNFs that did not meet with QIO staff either did not answer the QIO's initial phone calls or declined to participate, citing lack of time and competing priorities as their reasons." – [3]<br><br>"Leaders from all 5 SNFs expressed appreciation for the training and technical assistance provided by the QIO. However, they also confirmed QIO staff observations that staff at these SNFs were extremely busy with patient care and administrative duties and that it was difficult for them to find time for quality improvement." – [3]<br><br>"Operational manager unavailability due to other commitments delayed approval of improvement activities." [19]
Variable expertise and quality of supervision	"[…] the effectiveness of supportive supervision and mentoring from provincial and district levels was undermined by staff shortages at provincial and district levels and limited time spent at the health facilities. Providers noted that the quality of the coaching was often variable […] There were additional gaps in the quantity and quality of supervision and mentoring from the central level to the provinces and districts, which limited the ability of the districts to effectively coach the facilities." [14]<br><br>"In particular, there appears to have been variation in the quality of supervision and limitations around the delivery of the CQI training that likely contributed to the heterogenous results." [14]<br><br>"Greater monitoring of quality by district level authorities would help to bring about changes in practices such as postpartum monitoring and handwashing." [2]<br><br>"Further guidance for unit managers and staff in moving forward to develop micro-strategies could be provided, in future work, through educational sessions based on the PDSA model, the use of iterative cycles, the need for small-scale and prediction-based testing of change, the use of data over time, and the value and best practices for documentation" [16]<br><br>"The majority of programs reported staff problems that included time conflicts, where employees were unable to dedicate adequate time to the improvement efforts, lack of adherence to the strategies being implemented, lack of experience/training necessary to facilitate/implement components of planned programs, and lack of commitment to the program. " [20]
Centralized leadership	[…] fragmentation of quality assurance and improvement functions at national and provincial resulted in inefficiencies and ineffectiveness of interventions. Within the MHCC, quality improvement functions are distributed among different structural units with little coordination. This fragmentation is mirrored at lower levels such that CQI was perceived as another government program with dedicated people and resources, rather than a comprehensive and ingrained approach to quality improvement." [14]<br><br>"Macro and meso level strategies are likely needed at the national, provincial and district levels in addition to the micro level strategies to improve overall health system quality." [14]<br><br>"For both acceptability and sustainability, local leaders needed to spearhead the intervention. However, our direct involvement in driving the improvement work delayed the district managers accepting the intervention. This experience supports the suggestion from […] external assistance when developing QI approaches should focus on facilitation that supports local leadership to prioritise improvement projects and local health carer mentorship. [21]

	"While our results are promising, the extent to which the intervention has been sustained by the district managers is not known. It was made clear to the district managers up-front that the QI initiative would have external support from the project for 18 months, and thereafter they would need to take over implementation." [21]<br><br>"In our study, the surveillance was being driven by the principal investigator, who was not a staff member of any of the hospitals. Although feedback was provided at regular intervals on the quality of data being collected, it was evident that hospitals need to take ownership of the surveillance." [4]<br><br>"Feedback from some MDT members, however, indicated a lack of ownership of the decision to adopt MDT-FIT due to the centralized management of implementation." [1]
Communication and stakeholder relationships	"Six Sigma is a process that requires continuous reinforcement to be successful. Short-term goals can be easily achieved; however, gains made need to be sustained and the process needs to be continually monitored to ensure that old habits don't resurface." [17]<br><br>"Second, employee engagement and active participation should have been encouraged. Staff satisfaction and burnout with the project should have been tracked with a dedicated channel for staff to express their opinions and concerns about the project. Both horizontal and vertical buy-in should have been obtained." [17]<br><br>"Unfortunately, optimal results were not achieved due to participation issues, inefficient communication, and insufficient organizational buy-in." [17]<br><br>"Leadership was the second largest challenge for many. This included stakeholder relationships and cross-organizational partnerships where communication was minimal." [20]<br><br>"Although all 10 MDTs participated, this was perceived as mandatory by some, and often coupled with a lack of awareness of the potential benefits of participating." [1]<br><br>"There also appeared to be an "inverse" relationship between stakeholder inter-relationships and change infrastructure: the poor stakeholder interrelationships (due to lack of engagement of individual team members as implementation was centralized) led to some team members perceiving that MDT-FIT was mandatory rather than optional." [1]
Alignment of objectives and strategies	"The other major challenge that we anticipated was that decisions made by the multidisciplinary group could be in conflict with management plans made by attending neonatologists." [5]

References

1. Taylor C, Harris J, Stenner K, Sevdalis N, Green JSA. A multi-method evaluation of the implementation of a cancer teamwork assessment and feedback improvement programme (MDT-FIT) across a large integrated cancer system. Cancer Medicine. 2021;10(4):1240-52.
2. Iyengar K, Jain M, Thomas S, Dashora K, Liu W, Saini P, et al. Adherence to evidence based care practices for childbirth before and after a quality improvement intervention in health facilities of Rajasthan, India. BMC Pregnancy and Childbirth. 2014;14(1):270.
3. Meehan TP, Qazi DJ, Van Hoof TJ, Ho S-Y, Eckenrode S, Spenard A, et al. Process Evaluation of a Quality Improvement Project to Decrease Hospital Readmissions From Skilled Nursing Facilities. Journal of the American Medical Directors Association. 2015;16(8):648-53.
4. Mahomed S, Mahomed O, Sturm AW, Knight S, Moodley P. Challenges with Surveillance of Healthcare-Associated Infections in Intensive Care Units in South Africa. Critical Care Research and Practice. 2017;2017:7296317.
5. Welch CD, Check J, O'Shea MT. Improving care collaboration for NICU patients to decrease length of stay and readmission rate. BMJ Open Quality. 2017;6(2):e000130.
6. Chowdhury P, Tari A, Hill O, Shah A. To improve the communication between a community mental health team and its service users, their families and carers. BMJ Open Quality. 2020;9(4):e000914.
7. Doherty T, Chopra M, Nsibande D, Mngoma D. Improving the coverage of the PMTCT programme through a participatory quality improvement intervention in South Africa. BMC Public Health. 2009;9(1):406.
8. Brush JE, Balakrishnan SA, Brough J, Hartman C, Hines G, Liverman DP, et al. Implementation of a continuous quality improvement program for percutaneous coronary intervention and cardiac surgery at a large community hospital. American Heart Journal. 2006;152(2):379-85.
9. Rubenstein LV, Chaney EF, Ober S, Felker B, Sherman SE, Lanto A, et al. Using evidence-based quality improvement methods for translating depression collaborative care research into practice. Families, Systems, & Health. 2010;28:91-113.
10. Haraden C, Leitch J. Scotland's Successful National Approach To Improving Patient Safety In Acute Care. Health Affairs. 2011;30(4):755-63.
11. Radwan TF, Agyako Y EA, et al. Improving the management of type 2 diabetes through large-scale general practice: the role of a data-driven and technology-enabled education programme. BMJ Open Quality. 2021;10(1):e001087.
12. Davies C, Lyons C, Whyte R. Optimizing nursing time in a day care unit: Quality improvement using Lean Six Sigma methodology. International Journal for Quality in Health Care. 2019;31(Supplement_1):22-8.
13. Magge H, Kiflie A, Nimako K, Brooks K, Sodzi-Tettey S, Mobisson-Etuk N, et al. The Ethiopia healthcare quality initiative: design and initial lessons learned. International Journal for Quality in Health Care. 2019;31(10):G180-G6.
14. Gage AD, Gotsadze T, Seid E, Mutasa R, Friedman J. The influence of Continuous Quality Improvement on healthcare quality: A mixed-methods study from Zimbabwe. Social Science & Medicine. 2022;298:114831.
15. Mate KS, Ngubane G, Barker PM. A quality improvement model for the rapid scale-up of a program to prevent mother-to-child HIV transmission in South Africa. International Journal for Quality in Health Care. 2013;25(4):373-80.
16. Nyström ME, Höög E, Garvare R, Andersson Bäck M, Terris DD, Hansson J. Exploring the potential of a multi-level approach to improve capability for continuous organizational improvement and learning in a Swedish healthcare region. BMC Health Services Research. 2018;18(1):376.
17. Parikh N, Gargollo P, Granberg C. Improving Operating Room Efficiency Using the Six Sigma Methodology. Urology. 2021;154:141-7.

18. Waiswa P, Wanduru P, Okuga M, Kajjo D, Kwesiga D, Kalungi J, et al. Institutionalizing a Regional Model for Improving Quality of Newborn Care at Birth Across Hospitals in Eastern Uganda: A 4-Year Story. Global Health: Science and Practice. 2021;9(2):365.
19. Yapa HM, Dhlomo-Mphatswe W, Moshabela M, De Neve J-W, Herbst C, Jiamsakul A, et al. A Continuous Quality Improvement Intervention to Improve Antenatal HIV Care Testing in Rural South Africa: Evaluation of Implementation in a Real-World Setting. International Journal of Health Policy and Management. 2022;11(5):610-28.
20. Rocker GM, Amar C, Laframboise WL, Burns J, Verma JY. Spreading improvements for advanced COPD care through a Canadian Collaborative. International Journal of Chronic Obstructive Pulmonary Disease. 2017;12:2157-64.
21. Jaribu J, Penfold S, Green C, Manzi F, Schellenberg J. Improving Tanzanian childbirth service quality. International Journal of Health Care Quality Assurance. 2018;31(3):190-202.
