# Supplementary material for: Decision-maker roles in healthcare quality improvement projects: a scoping review
Source: BMJ Open Qual. 2024 Jan 5;13(1):e002522. doi: 10.1136/bmjoq-2023-002522 (PMC10773379; doi:10.1136/bmjoq-2023-002522)
Supplement: Supplementary data [file bmjoq-2023-002522supp002.pdf]

Appendix II. Expanded Tables Displaying the Extracted Data

Table 1. QI project characteristics (expanded)

Authors	Country	How QI is documented	Setting	Problem	Scope	Improvement Framework	QI Objective	Change Strategies	Observed Change	Decision-Makers Involved
Arbour et al. 2021	United States	Retrospective description of QI program and outcomes	Primary care (5)	Maternal depression, intimate partner violence (IPV), and social needs	Multi	PDSA cycles	In five sites, 75% of infants and their families will receive all recommended well-child visits on time	DULCE (Developmental Understanding and Legal Collaboration for Everyone):<br>• Linking families with infants to needed resources	• High proportion of families screened for health-related social needs<br>• High proportion of families provided information about resources<br>• Increase in the percentage of families who completed well-child visits on time	Regional:<br>• Not specified<br>Local:<br>• Clinic administrator
Brush et al. 2006	United states	Retrospective description of QI program and outcomes	Hospital (1)	Suboptimal cardiac surgery outcomes	Single	None described	Improve cardiac surgery outcomes through practice standardization	• Data for percutaneous coronary intervention and cardiac surgery are collected<br>• Performance reports are reviewed and methods for improving performance are implemented	• Improvements in several cardiac surgery indicators	Local:<br>• Administrators
Chowdhury et al. 2020	United Kingdom	Retrospective description of QI program and outcomes	Specialized (1)<br>• Community mental health team	Service users or their carers feel they are not given enough information	Single	PDSA cycles	Improve communication between mental health team and service users, families, and carers	• Medication leaflets for clinicians,<br>• Diagnostic leaflets for clinicians<br>• Amendment of clinician letters<br>• Mandatory diary updating<br>• Structured information entry	• Increase in service user satisfaction<br>• Reduction in complaints	Local:<br>• Team manager<br>• Deputy manager<br>• Team administrative manager
Davies et al. 2019	Ireland	Retrospective description of QI program and outcomes	Hospital (1)	Inefficiencies in nursing care and suboptimal work conditions	Single	Lean Six Sigma (LSS)	Optimize nursing time and improve personalized care and staff satisfaction	• Streamlining documentation<br>• Visual indicator for refreshment needs,<br>• Increasing patient capacity<br>• Team-building	• Service performance<br>• Patient satisfaction<br>• Staff satisfaction.<br>• Reduction in patient turnaround time<br>• Increase in nursing care time<br>• Improvement of nurse-patient ratio	Local:<br>• Hospital CEO<br>• Director of Nursing
Doherty et al. 2009	South Africa	Retrospective description of QI program and outcomes	Multiple institutions in one district	High rates of mother-to-child HIV transmission	Multi	None described	Scale up program to prevent mother-to-child HIV transmission	PMTCT (prevent mother-to-child transmission) programme:<br>• Routine offer of antenatal counselling and testing<br>• Infant feeding counselling<br>• Nevirapine for mothers and infants	• Increase in rate of antenatal and PCR testing<br>• Uptake of infant nevirapine	Regional:<br>• District programme managers<br>Local:<br>• Unit managers<br>• Clinic supervisors

								• Infant PCR testing<br>• Free formula for women choosing not to breastfeed		
Gage et al. 2022	Zimbabwe	Retrospective description of QI program and outcomes and factors associated with implementati on	Multiple institutions (30) in 5 districts<br>• Primary care<br>• Hospitals	Suboptimal maternal, newborn and child health services	Multi	PDSA cycles	Improve the quality of maternal, newborn, and child health services	Capacity building to conduct QI	• Improvement in postnatal care process measures<br>• Improvement in maternal care process measures	Regional:<br>• District managers<br>• Ministry of Health and Child Care<br><br>Local:<br>• Managers
Gerrish et al. 2018	United Kingdom	Retrospective description of QI program and outcomes and factors associated with implementati on	Multiple institutions<br>• Primary care<br>• Hospitals<br>• Other service providers	Risk of falls among elderly population and fall-related injury	Multi	Clinical micro-systems approach	Improve quality of care within the falls pathway	Integrated care pathway for falls:<br>• Screening<br>• Risk assessment<br>• Multidisciplinary diagnosis of unexplained falls<br><br>• Patient information<br>• Enhanced data collection<br>• Improved staff communication, coordination and role clarification<br>• Referral process redesign	• Optimization of various aspects of the pathway<br>• Collaboration between frontline staff and decision-makers from different services within the pathway was achieved	Regional:<br>• Not specified<br><br>Local:<br>• Clinical managers<br>• Senior clinical managers<br>• Service managers
Haraden et al. 2011	Scotland	Description of QI program and intermediate outcomes	Hospital (9)	High rate of surgical mortality	Multi	Model for Improvement (PDSA cycles)	Improve care quality (person-centered, safe and effective) for acute care patients	Capacity building to conduct QI	• Decrease in mortality rate	Regional:<br>• Clinical managers<br>• Local health boards<br>• Government<br><br>Local:<br>• Managers
Hill et al. 2015	United States	Retrospective description of QI program and outcomes	Hospital (1)	High rate of staff injury on inpatient child/adolescent psychiatric unit	Single	• Intermediate Improvement Science Series (I2S2)<br>• System of profound knowledge<br>• PDSA cycles	Reduce staff injury related to patient interactions in inpatient child/adolescent psychiatric unit	• Precautionary measures<br>• Standardized assessment<br>• Protective equipment<br>• Team huddles<br>• Leadership rounds<br>• Incident reviews<br>• Safe handoff<br>• Patient information binders<br>• Patient identification board	• Reduction in injuries	Local:<br>• Nursing director
Iyengar et al. 2014	India	Retrospective description of QI program and outcomes	Multiple institutions in 10 districts<br>• Public health facilities (44)	Gaps in the quality of childbirth services for institutional deliveries	Multi	None described	Improve quality of childbirth services public service facilities	• Capacity building to conduct QI<br>• Evidence based practice recommendations	• Reduction of unnecessary or harmful practices during labor, delivery and postpartum (in most facilities)	Regional:<br>• State government<br>• District health officers and State Managers

								• Staff received training		Local:<br>• Not specified
Jaribu et al. 2015	Tanzania	Retrospective description of QI program and outcomes	Multiple institutions (27) in 2 regions<br>• Health centres<br>• Dispensaries	Elevated maternal and neonatal morbidity and mortality	Multi	• Breakthrough series collaborative<br>• (PDSA cycles)	Increase the rate of facility-based deliveries and improve the quality of perinatal care	• Improved monitoring and detection of problems at childbirth<br>• Counselling of pregnant women	• Increase in facility-based deliveries<br>• Increase in number of partographs to monitor labor progress	Regional:<br>• District managers<br>Local:<br>• Not specified
Magge et al. 2019	Ethiopia	Description of QI program and intermediate outcomes and QI implementati on measures	Multiple institutions in four regions within in one district<br>• Public health facilities	Elevated rate of maternal and infant mortality	Multi	PDSA cycles	Improve maternal and child health and reduce maternal and infant mortality	• National quality strategy<br>• QI collaborative infrastructure<br>• Capacity building to conduct maternal and newborn health QI	• Numerous maternal and newborn health change ideas were tested<br>• A change package was developed with 83 successfully tested change ideas	Regional:<br>• Federal Ministry of Health<br>• Regional Health Bureaus<br>Local:<br>• Facility managers
Mahomed et al. 2017	South Africa	Retrospective description of QI program and outcomes and factors associated with implementati on	Hospital (8)<br>• Intensive Care Units	High rate of healthcare associated infections	Multi	PDSA cycles	Implement a paper-based surveillance system for ICUs to measure healthcare-associated infections	Surveillance system for monitoring and preventing healthcare-associated infections in ICUs	• Surveillance system implementation faced numerous challenges and was not successfully achieved	Regional:<br>• Department of Health Provincial Infection Prevention and Control Unit<br>Local:<br>• Senior management<br>• ICU nursing manager<br>• Clinical manager
Mate et al. 2013	South Africa	Retrospective description of QI program and outcomes	Multiple institutions (161) in 18 health districts<br>• Health care facilities	High rate of mother-to-child HIV transmission	Multi	Model for improvement (PDSA cycles)	Improve the quality of antenatal care and prevent mother-to-child HIV transmission	A-Plan (Accelerated plan):<br>• Increase the number of pregnant women in antenatal care<br>• Strengthen preventative services at health-care facilities	• Tracking of processes in the prevent mother-to-child transmission program was established	Regional:<br>• National Department of Health<br>• District managers<br>Local:<br>• Facility managers
Meehan et al. 2015	United States	Retrospective description of QI program and outcomes and factors associated with implementati on	Specialized facilities (5)<br>• Skilled nursing facilities	High rate of hospital readmissions within 30 days post-discharge	Multi	None described	Decrease the rate of avoidable hospital readmissions	INTERACT (Interventions to Reduce Acute Care Transfer):<br>• Improve identification, evaluation, and management of acute changes in residents' conditions<br>• Implement tools to support these aims	• INTERACT tools implemented in all facilities<br>• Hospital readmission rates decreased in two facilities	Regional:<br>• Not specified<br>Local:<br>• SNF Administrator<br>• Director of Nursing
Needleman et al. 2016	United States	Retrospective description of QI program and QI implementati on measures	Hospital (67)	Suboptimal safety, reliability and patient-centredness of inpatient care	Multi	PDSA cycles	Improve quality and efficiency of inpatient care and support more effective teamwork	• QI collaborative infrastructure<br>• QI capacity building	• Aspects of the QI project implemented in all participating hospitals<br>• A large proportion of teams succeeded in conducting tests of change	Local:<br>• Unit managers<br>• Nursing administrators<br>• Physician administrators<br>• Department heads
Nyström et al. 2018	Sweden	Retrospective description of QI program and QI implementati on measures	Specialized facilities in two municipalities<br>• Specialized care residences	Suboptimal elder care and care for children with functional impairments	Multi	SIDSSA (development and action-learning loops)	Improve care knowledge and capabilities of service providers	• Multi-level QI capacity building strategy	• Aspects of the QI intervention implemented in the participating settings<br>• Participants' perceptions of the approach were favorable	Regional:<br>• Division managers<br>Local:<br>• Unit managers

Parikh et al. 2021	United States	Retrospective description of QI program and outcomes	Hospital (1)	Inefficiencies in ambulatory surgery	Single	Six Sigma (DMAIC)	Increase patient satisfaction, improve quality of care, and increase efficiency of patient flow related to ambulatory surgery	• Better define and optimize staff roles<br>• Improve communication between operating room staff	• Some (but not all anticipated) improvements to workflow were observed	Local:<br>• Administrators
Radwan et al. 2020	United Kingdom	Retrospective description of QI program and outcomes	Primary care (1)	Inequity in the diabetes care and diabetes outcomes	Single	PDSA cycles	Improve measurable diabetes-clinical outcomes in marginalized ethnic communities	• Information gathering<br>• Pharmacist education<br>• Process optimization	Outcomes<br>• Improvements to care processes<br>• All treatment targets for patients with diabetes	Local:<br>• Managers
Raman et al. 2022	United States	Retrospective description of QI program and outcomes	Hospital (1)<br>• Pediatric hospital	Inefficiencies in adolescent scoliosis operations	Single	None described	Improve perioperative efficiency in adolescent idiopathic scoliosis	CUSP (Comprehensive Unit-based Safety Program)<br>• Increasing visibility of metrics for duration<br>• Partnering with blood bank<br>• Streamlining OR trays<br>• Improving of OR setup and staffing	Outcomes<br>• Increase in first case on-time start<br>• Variance decrease for anesthesia ready time<br>• Decrease in closure to patient out of room time	Local:<br>• Senior executives<br>• RN managers
Read Sermersheim et al. 2021	United States	Retrospective description of QI program and outcomes	Hospital (1)	High incidence of hospital acquired injuries related to devices placed around the nares	Single	"RUSH Way" (combines PDCA, Six Sigma, Lean)	Reduce the incidence of nares acquired pressure injuries (NAPIs) to 3%	Evidence-based nares acquired pressure injuries (NAPIs) preventive care bundle comprising an improved tube fastening system<br>• hydrocolloid barrier<br>• tube securement device<br>• patient assessments<br>• site checks	Outcomes<br>• Reduction in nares acquired pressure injuries incidence rate	Local:<br>• Manager from nursing department
Rocker et al. 2017	Canada	Retrospective description of QI program and outcomes and QI implementation measures	Multiple institutions (78) across 10 provinces<br>• Acute care<br>• Primary care<br>• Home care	Suboptimal COPD care and burden of disease for patients and health systems	Multi	PDSA cycles	Improve COPD care quality and patient-centeredness	INSPIRED COPD Outreach Program:<br>• Post-discharge follow-up<br>• Psychosocial support<br>• Access to support services	• Aspects of the QI intervention implemented to varying degrees<br>• Many teams involved patients in customized care planning<br>• Admissions, emergency room visits and costs decreased in some facilities<br>• Teams reported gaining greater knowledge about COPD care	Regional:<br>• Not specified<br>Local:<br>• Nurse managers
Rubenstein et al. 2010	United States	Description of QI program and	Primary care (3) in three regions	Suboptimal quality of depression care	Multi	PDSA cycles	Implement research-based collaborative	Translating Initiatives in Depression into Effective Solution (TIDES):<br>• Pathway	Outcomes<br>• Decrease in mean Patient Health Questionnaire (PHQ-9) scores	Regional:<br>• Regional director<br>Local:

		intermediate outcomes					care for depression	• Manager roles<br>• Clinician roles<br>• Informatics<br>• Education	• Care managers referred 28% of TIDES patients to mental health specialty	• Clinical and administrative leaders
Shi et al. 2022	Taiwan	Retrospective description of QI program and outcomes	Hospital (1)	Elevated rate of surgical site infections	Single	Six Sigma (DMAIC)	Reduce the surgical site rates by 20%	• Strengthen leadership support system<br>• Perioperative risk assessment<br>• Standardize perioperative protocols<br>• Healthcare information feedback system<br>• Environmental cleaning audit system	• Decrease in surgical site infections	Local:<br>• Managers of the general affairs office<br>• Dean and hospital administrators
Taylor et al. 2021	United Kingdom	Retrospective description of QI program and outcomes QI implementati on measures	Multiple institutions (10)<br>• Integrated Care Centres	Suboptimal breast cancer care	Multi	None described	Improve multi disciplinary team breast cancer care	Multi Disciplinary Team (MDT) Fit Program:<br>• Assessment and feedback programme	• All teams within the care centers participated in the implementation of MDT-FIT<br><br>• Moderate fidelity<br>• High acceptability<br>• Moderate adoption<br>• High appropriateness<br>• Low cost<br>• High feasibility	Regional:<br>• Integrated care system (ICS) director<br><br>Local:<br>• Managers<br>• Administrators
Villarreal et al. 2015	United States	Retrospective description of QI program and outcomes	Hospital (1)<br>• Radiology Department	Delays in procedure start times in radiology	Single	None described	Improve efficiency and reduce delays in radiology start times	• Checklist for treatment room handoff<br>• Redefinition of transport responsibility<br>• Timing of blood draw modified<br>• Consent performed by fellows<br>• Lab results made accessible via internet	• Increase in number of on-time starts<br>• Reduction in patient wait times<br>• Reduction in delay duration<br>• Reduction in subsequent patient care delays	Local:<br>• Manager<br>• Nurse manager
Waiswa et al. 2021	Uganda	Retrospective description of QI program and outcomes	Hospital (6)	High rate of adverse birth outcomes	Multi	PDSA cycles	Improve the quality of maternal and neonatal care in hospitals	Standardized package that included:<br>• Training<br>• equipment and data collection<br>• clinical mentorship<br>• Support for improved clinical audits<br>• Support for continuous leadership engagement	• Neonatal Care Units in maternity units institutionalized, as well as kangaroo mother care spaces, resuscitation corners, and MPDRs in the maternity units<br><br>• Reduction in maternal and neonatal mortality rates	Regional:<br>• Health system managers<br>• District health officers<br>• Minister of Health Obstetricians<br><br>Local:<br>• Medical superintendents<br>• hospital administrators<br>• Neonatal care unit managers
Welch et al. 2017	United States	Retrospective description of QI program and outcomes	Hospital (1)	Burden of medically complex patients in neonatal intensive care units, and diminished collaboration and care continuity	Single	PDSA cycles	Improve collaboration and continuity of care, decrease length of stay, and improve parent satisfaction	Care Collaboration for Babies with Extended Stays (CBES):<br>• Weekly multidisciplinary team meetings to discuss long term plans for patients	Outcomes<br>• Reduction in duration of hospitalization	Local:<br>• NICU Medical director<br>• NICU Nurse manager

Yapa et al. 2019	South Africa	Retrospective description of QI processes and outcomes and factors associated with implementati on	Primary care (7)	High rate of mother-to-child HIV transmission	Multi	PDSA cycles	Improve Antenatal HIV care	MONARCH (Management and Optimisation of Nutrition, Antenatal, Reproductive, Child health and HIV care):<br>• Viral load monitoring among pregnant women living with HIV<br>• Repeat testing among women not living with HIV	• CQI not fully implemented and normalized<br>• Patient tracking notebook and results filing system implemented<br>• Viral load monitoring improved	Regional:<br>• Not specified<br>Local:<br>• Clinic operational managers

Table 2. The roles decision-makers played in the QI projects (expanded)

Authors	QI project Initiator	Local QI project leader(s)	Decision-maker level	Decision-maker role	Decision-makers' documented contributions	Observed benefits of decision-maker involvement	Observed challenges with decision-maker involvement
Arbour et al. 2021	Non-government organization<br>• Center for the Study of Social Policy (CSSP)	Local QI team	Regional	Insufficient detail	Not specified	None described	None described
			Local	Collaborator	Clinic administrator:<br>• Member of local implementation team		
Brush et al. 2006	Local actor(s)<br>• Data committee<br>• Quality committee	Local QI team<br>• Data committee<br>• Quality committee	Local	Collaborator	Administrators:<br>• Member of Data Committee and Quality Committee<br>• Responsibilities included launching, coordinating, and institutionalizing QI methods<br>• Quality committee initiated and led the project.	• Collaboration with decision-makers promoted a sustained spirit of cooperation<br>• Collaboration supported shared learning, leadership and problem solving<br>• Decision-maker involvement helped gain physician buy-in	None described
Chowdhury et al. 2020	Not specified	Local Decision-Maker<br>• Team manager	Local	Collaborator	Team manager, deputy manager, and team administrative manager:<br>• Weekly meetings to brainstorm ideas, plan tests of change, review progress and define courses of action<br>• The team measured baseline data, established the design and theory change and participated in PDSA cycles.	None described	None described
Davies et al. 2019	Not specified	Local QI team<br>• Nursing division staff	Local	Hospital CEO<br>• Supporter<br>• Consultant<br><br>Director of Nursing<br>• Supporter<br>• Consultant	Hospital CEO<br>• Project sponsor<br>• Consulted for defining the strategy<br><br>Director of Nursing<br>• Project champion<br>• Consulted for defining the strategy	• Senior management involvement and financial investment considered crucial	None described
Doherty et al. 2009	Regional team (including regional decision-makers)<br>• Programme managers<br>• Unit managers<br>• Clinic supervisors	Regional QI teams<br>• District and sub-district coordinators<br>• Supervisors	Regional	Collaborator	District programme managers<br>• Participated in the assessment phase task team<br>• Participated in training workshops and feedback sessions<br>• Gained skills in programme assessment<br>• Helped identify gaps and define goals and action plans<br>• Participated in implementation and monitoring	• Participatory approach enabled mid level managers to see how their facilities functioned and take ownership	• Support from senior district management critical to enabling middle management participation
			Local	Collaborator	Unit managers and clinic supervisors<br>• Participated in the assessment phase task team<br>• Participated in training workshops and feedback sessions<br>• Helped identify gaps and define goals and action plans<br>• Participated in implementation and monitoring		
Gage et al. 2022	Regional decision-maker<br>• Ministry of Health and Child Care	Local QI team<br>• CQI teams	Regional	Ministry of Health and Child Care<br>• Initiator<br><br>District managers<br>• Supporter	Ministry of Health and Child Care<br>• Initiated the national program which comprised the QI pilot<br>• Made QI a priority<br><br>District managers	• Leadership, teamwork and joint decision-making considered enabling factors<br>• Supportive supervision from district authority was considered motivating	• Some participants viewed the QI project as a top-down intervention<br>• Quality of supervision and mentoring varied between districts

					• Trained and supported to implement QI		• National QI projects require strategies at all levels (macro, meso and micro)
			Local	Collaborator	Managers<br>• Received coaching to carry out QI activities<br>• Led the program within each of the facilities<br>• Consulted and engaged staff in identifying factors, monitoring results, and decision making		
Gerrish et al. 2018	Non-government organization<br>• Teaching Hospitals and healthcare partners	Local QI team<br>• Nurse<br>• Facilitators	Regional	Insufficient detail	Not specified	None described	None described
			Local	Collaborator	Clinical managers<br>• Involved in meso level group for the change initiation phase<br>• Involved in macro level group for the achieving change phase<br><br>Service managers<br>• Involved in macro level group for the achieving change phase		
Haraden et al. 2011	Regional decision-maker<br>• Government health directorate	Local decision-maker<br>• Program manager<br>• Executive leader	Regional	Local health boards<br>• Supporter<br><br>Scottish government<br>• Initiator	Local health boards<br>• Recruited to get clinician buy-in.<br>• Participated in leadership walk-arounds<br><br>Scottish government<br>• Worked with IHI on a measurement plan to gather data for run charts	• Leadership involvement considered key to clinician buy-in and local initiatives being adequately supported	None described
			Local	Collaborator	Managers<br>• Received QI training alongside leaders and clinicians<br>• Collaborated with government on aligning goals<br>• Led local QI projects		
Hill et al. 2015	Not specified	Local QI team<br>• Project team	Local	Collaborator	Nursing director<br>• Member of QI team<br>• The team established the goals, collected and interpreted data, identified and implemented strategies	None described	None described
Iyengar et al. 2014	Regional team (including regional decision-makers)<br>• State Government<br><br>• Action Research & Training for Health<br>• United Nations Population Fund	Not specified	Regional	• Initiator<br>• Supporter	State Government Medical, Health & Family Welfare Department<br>• Collaborated with partners on identifying facilities and developing the initiative<br>• Sent letters of invitation to participating facilities<br>• Participated in workshops on the role of evidence based care<br><br>District health officers and State Managers<br>• Received report cards and attended annual review meetings<br>• Participated in workshops on the role of evidence based care	• Close collaboration with state and district level decision makers from the design stage considered crucial to building consensus and sustaining QI	• Better monitoring by district authority would have better supported practice change
			Local	Insufficient detail	Not specified		
Jaribu et al. 2015	Regional decision-maker<br>• Ministry of health Health, and Social Welfare<br>• Mtwara Rural and Ruangwa Council Health	QI Facilitator	Regional	Collaborator	District managers<br>• Participated in QI workshops<br>• Included as "collaborators"	None described	• Directives from district managers and local managers' limited influence hindered acceptability and sustainability
			Local	Insufficient detail	Not specified		

	Management Teams (CHMT)<br>• Ifakara Health Institute (IHI) staff<br>• Improving New-born Survival in Southern Tanzania (INSIST) team						• District managers must support local managers prioritize QI<br>• District managers received external support for 18 months then support was withdrawn
Magge et al. 2019	Regional decision-maker<br>• Ministry of Health<br>• Institute for Healthcare Improvement (IHI)	Local QI team<br>• Multi disciplinary teams	Regional	Initiator	Federal Ministry of Health<br>• Partnered with the Institute for Healthcare Improvement (IHI) to identify opportunities for improvement<br>• Consulted regarding the selection of five prototype collaboratives<br>• Initiated and planned the initiative at the national level	• The Minister of Health's leadership in the design and implementation was considered a major enabling factor<br>• Ministry of health investment and incorporation of the QI strategy into central policy considered key<br>• Learning sessions helped link health system actors with community members to advance patient-centered care	None described
			Local	Collaborator	Facility managers<br>• Served as members on each of the collaboratives' multidisciplinary teams<br>• Assisted with the development of action plans to addressed gaps including clinical training		
Mahomed et al. 2017	Researchers	Researchers	Regional	Supporter	Department of Health Provincial Infection Prevention and Control<br>Unit and senior management<br>• Provided support to the ICUs in their conduct of QI	None described	• Local managers did not take ownership of the process nor maintain adequate oversight<br>• Nursing managers were often occupied with their other duties
			Local	Senior management, clinical manager<br>• Consultant<br>Nursing manager<br>• Consultant<br>• Supporter	Senior management<br>• Consulted for planning<br>• Participated in review meeting to decide if the surveillance system should be adopted<br>Nursing manager<br>• Consulted for planning<br>• Approved the tool prior to implementation<br>• Provided feedback from evaluation of the surveillance system<br>• Participated in review meeting to decide if the surveillance system should be adopted<br>• Ensured the night staff received training<br>Clinical manager<br>• Consulted for planning<br>• Met with principle investigator to review patient charts<br>• Approved the tool prior to implementation<br>• Participated in review meeting to decide if the surveillance system should be adopted		
Mate et al. 2013	Regional decision-maker<br>• South Africa National Department of Health<br>• Prevention of mother-to child HIV transmission (PMTCT) Directorate	Project leader	Regional	National Department of Health<br>• Initiator<br>District managers<br>• Supporter	National Department of Health<br>• Initiated the national strategy<br>District managers<br>• Designated NGOs to participate<br>• Planned the QI project<br>• Participated in monthly district review meetings	• District managers' involvement at early stages and project leadership considered key success factors<br>• Ownership by National department of health leaders was considered essential	None explicitly discussed
			Local	Insufficient detail	Facility managers<br>• Involved in local execution of the QI project		

Meehan et al. 2015	Non-government organization<br>• Connecticut Quality Improvement Organization	QI Facilitators	Regional	Insufficient detail	Not described	None explicitly discussed	• Many leaders approached declined to participate due to lack of time
			Local	Supporter	Administrators and nursing managers<br>• Asked to give their support<br>• Some attended QI training sessions<br>• Offered technical assistance		
Needleman et al. 2016	Non-government organization<br>• Robert Wood Johnson Foundation (RWJF)<br>• American Organization of Nurse Executives (AONE)	Local QI team<br>• Nursing unit	Regional	Insufficient detail	Not described	None explicitly discussed	• Leaders were encouraged to create hospital level teams to support QI teams, but they did in less than a third of sites
			Local	Unit managers<br>• Collaborator<br><br>Other decision-makers<br>• Collaborator (some sites)	Unit managers<br>• Attended or led the regular meetings<br>• Some were also members of a leadership team that discussed reports from unit managers, tests of change and sustainability<br><br>Nursing administrators, physician administrators, department heads<br>• Participated in hospital level teams (some sites)<br>• Member of leadership team that discussed reports from unit managers, tests of change and sustainability (some sites)		
Nyström et al. 2018	Regional team (including regional decision-makers)<br>• Researchers<br>• Public health agency	Local decision-maker<br>• Division managers<br>• Unit managers	Regional	Collaborator	Division managers<br>• Participated in meetings and workshops<br>• Responsible for overseeing change and aiding staff with implementation	• Strategic management involvement, coaching, and working with managerial colleagues enhanced the observed change and learning processes	• Managers could have benefitted from guidance regarding micro-strategies<br>• When involvement of division managers decreased, progress slowed
			Local	Collaborator	Unit managers<br>• Participated in meetings and workshops<br>• Locally responsible for overseeing change and aiding staff with implementation		
Parikh et al. 2021	Not specified	Local decision-maker<br>• Administrators	Local	Collaborator	Administrators<br>• Member of the leadership team	• Leadership involving each stakeholder considered success factor	• Lack of continuous reinforcement contributed to resistance and slowed progress<br>• Participation issues, inefficient communication, and insufficient organizational buy-in
Radwan et al. 2020	Regional decision-maker<br>• National Health Service	Local QI team<br>• Multi-disciplinary teams	Local	Collaborator	Managers<br>• Co-designed interventions<br>• Participated in QI training program<br>• Member of the implementation team<br>• Facilitated recalls and reviews to be undertaken by clinical staff	• Managers and staff played a role, giving the whole team a sense of ownership	None explicitly discussed
Raman et al. 2022	Local actor(s)<br>• Attending surgeon	QI Facilitator	Local	Collaborator	Senior executives and nurse managers<br>• Provided training<br>• Attended regular meetings<br>• Assisted with the alignment of goals, assessment of needs and development of interventions	None explicitly discussed	None explicitly described
Read Sermershei m et al. 2021	Local actor(s)<br>• QI team	Local QI team	Local	Collaborator	Nursing manager<br>• Member of the QI team<br>• Co-designed the project charter and plan<br>• Participated in practice evaluation and identification of gaps	None explicitly discussed	None explicitly described

					• Helped ensure that education was institutionalized		
Rocker et al. 2017	Non-government organization<br>• Foundation for Healthcare Improvement	Local QI team<br>• QI team<br>• Foundation for Healthcare Improvement	Regional	Insufficient detail	Not specified	None explicitly discussed	• Lack of experience and training hindered optimal facilitation and implementation of program components<br>• Benefits of the collaborative not fully realized due to minimal communication between leadership and partner organizations
			Local	Collaborator	Nurse managers<br>• Members of local QI teams		
Rubenstein et al. 2010	Researchers	Researchers	Regional	Collaborator	Regional director<br>• Endorsed the project<br>• Reviewed collaborative care literature provided by the research team<br>• Participated in the design of the intervention as member of design panel<br>• Participated in PDSA cycles	• Support from primary care and mental health leadership at all levels considered critical<br>• Buy-in from managers responsible for resource allocation considered critical	• Leadership involvement did not persist
			Local	Collaborator	Clinical and administrative leaders<br>• Implemented the interventions<br>• Participated in PDSA cycles		
Shi et al. 2022	Local actor(s)<br>• QI team	Local QI team	Local	Managers at the general affairs office<br>• Collaborator<br><br>Dean and hospital administrators<br>• Supporter	Managers at the general affairs office<br>• Member of the QI team<br><br>Dean and hospital administrators<br>• Approved and enforced the policies and improvement processes<br>• Supported implementation<br>• Provided the necessary resources	None explicitly discussed	None explicitly described
Taylor et al. 2021	Regional decision-makers<br>• Integrated care system (ICS) management	Local decision-maker<br>• Integrated care system (ICS) administrator<br>• Cancer managers<br>• Hospital administrators	Regional	• Initiator<br>• Supporter	Integrated care system (ICS) administrator<br>• Coordinated hospital cancer managers and administrators<br>• Introduced the program to local teams<br>• Received weekly updates from the teams	None explicitly discussed	• Poor stakeholder relationships resulted in a more centralized approach to implementation<br>• Centralized approach hindered a sense of ownership<br>• The choice by the ICS not to engage hospital based managers and administrators led to a staggered implementation
			Local	• Initiator<br>• Supporter	Managers and administrators<br>• Introduced the program<br>• Identified project champions<br>• Received weekly updates		
Villarreal et al. 2015	Local actor(s)<br>• Radiology department	QI Facilitator<br>• Vice chair of quality	Local	Collaborator	Manager and nurse manager<br>• Member of the QI team<br>• Participated in mapping patient workflow and defining metrics<br>• Planned and implemented interventions	• Interdisciplinary multi-level approach provided complimentary insight and supported buy-in at different levels	None explicitly discussed
Waiswa et al. 2021	Non-government organization<br>• The World Health Organization (WHO)	Local QI team<br>• Hospital teams led by maternity nurse in charge	Regional	Collaborator	Health system managers, District health officers, Minister of Health Obstetricians<br>• Participated in health system managers meetings<br>• Co-designed the intervention	• Collaboration between facilities helped establish a regional care network	None explicitly discussed

					• Reviewed findings from baseline survey and identified priority interventions for implementation<br>• Participated in the development, implementation, and evaluation of the project interventions	• Involvement of managers helped ensure feasibility as they knew what would work in their facilities<br>• Managers' endorsement of interventions helped ensure their successful implementation	
			Local	Collaborator	Medical superintendents, hospital administrators, unit managers<br>• Participated in health system managers meetings<br>• Co-designed the intervention<br>• Reviewed findings from baseline survey and identified priority interventions for implementation<br>• Assisted with the implementation		
Welch et al. 2017	Local actor(s)<br>• Clinician researchers	Local QI team	Local	Medical director of the NICU<br>• Consultant<br><br>NICU nurse manager<br>• Collaborator	Medical director of the NICU<br>• Consulted on the intervention and primary outcomes<br><br>NICU nurse manager<br>• Participated in multidisciplinary team meetings (clinical and QI)<br>• Collaborated on the development of the intervention<br>• Discussed interim results and planned and implemented modifications	None explicitly discussed	• QI team's decisions can conflict with manager's plans
Yapa et al. 2019	Non-government organizations<br>• NHS foundation trust<br><br>• Clinicians<br>• Researchers<br>• QI facilitators	QI Facilitators	Regional	Insufficient detail	Not specified	• Involvement of the operational manager considered essential for authorizing activity	• Time commitments limited operational manager involvement and subsequently delayed improvement activities
			Local	Collaborator	Clinic operational managers<br>• Guided clinic teams within each facility to identify areas for improvement and test solutions<br>• Attended CQI team meetings in some settings		
